# Nitrated Nucleotides: New Players in Signaling Pathways of Reactive Nitrogen and Oxygen Species in Plants

**DOI:** 10.3389/fpls.2020.00598

**Published:** 2020-05-19

**Authors:** Marek Petřivalský, Lenka Luhová

**Affiliations:** Department of Biochemistry, Faculty of Science, Palacký University, Olomouc, Czechia

**Keywords:** nitration, nitric oxide, plant, persulfidation, reactive nitrogen species, reactive oxygen species, signaling, S-guanylation

## Abstract

Nitration of diverse biomolecules, including proteins, lipids and nucleic acid, by reactive nitrogen species represents one of the key mechanisms mediating nitric oxide (NO) biological activity across all types of organisms. 8-nitroguanosine 3′5′-cyclic monophosphate (8-nitro-cGMP) has been described as a unique electrophilic intermediate involved in intracellular redox signaling. In animal cells, 8-nitro-cGMP is formed from guanosine-5′-triphosphate by a combined action of reactive nitrogen (RNS) and oxygen species (ROS) and guanylate cyclase. As demonstrated originally in animal models, 8-nitro-cGMP shows certain biological activities closely resembling its analog cGMP; however, its regulatory functions are mediated mainly by its electrophilic properties and chemical interactions with protein thiols resulting in a novel protein post-translational modification termed S-guanylation. In *Arabidopsis thaliana*, 8-nitro-cGMP was reported to mediate NO-dependent signaling pathways controlling abscisic acid (ABA)-induced stomatal closure, however, its derivative 8-mercapto-cGMP (8-SH-cGMP) was later shown as the active component of hydrogen sulfide (H_2_S)-mediated guard cell signaling. Here we present a survey of current knowledge on biosynthesis, metabolism and biological activities of nitrated nucleotides with special attention to described and proposed functions of 8-nitro-cGMP and its metabolites in plant physiology and stress responses.

## Introduction

Nitric oxide (NO) is a crucial gaseous signaling molecule which plays vital roles in a broad spectrum of physiological and developmental processes throughout the plant life, including germination, development of leaves, roots and reproductive organs, stomata movement and plant senescence (Yu et al., 2014; Astier et al., 2018). NO also participates in signaling pathways of plant reactions to biotic and abiotic stresses (Corpas and Barroso, 2015; Farnese et al., 2016). Signaling functions of NO in plants are mediated namely through three key NO-dependent post-translational modifications (PTM): metal nitrosylation in metalloproteins, S-nitrosation of cysteine thiols, and tyrosine nitration (Begara-Morales et al., 2016; Umbreen et al., 2018). Nitration of proteins, lipids, nucleic acid and free nucleotides occurs in plants as a part of NO-dependent signaling pathways within redox regulations and during plant responses to diverse environmental stress stimuli (reviewed in Arasimowicz-Jelonek and Floryszak-Wieczorek, 2019). Here we present a concise overview of current knowledge on formation, metabolism and biological activities of nitrated nucleotides with special attention to described and proposed functions of 8-nitro-cGMP and its metabolites in plants.

## Formation of Nitrated Nucleotides

The possibility of covalent modification of nucleotides and related compounds has been studied since the early years of NO research in animals. 8-Nitroguanine was reported to originate in a dose-dependent manner from a rapid reaction of peroxynitrite with free guanine nucleotide (Yermilov et al., 1995a) as well with guanine in calf thymus and epidermal keratinocytes DNA *in vitro* (Yermilov et al., 1995b; Spencer et al., 1996). *In vivo* formation of 8-nitroguanine and related nitrated derivatives was reported in livers of hamsters after infection with *Opisthorchis viverrini* (Pinlaor et al., 2003) and in human gastric mucosa upon *H. pylori* infection (Ma et al., 2004). Guanosine can be also readily nitrated by reactive nitrogen species *in vitro* (Niles et al., 2001; Sodum and Fiala, 2001). 8-Nitroguanosine formation occurred in RNA of peroxynitrite-treated human lung carcinoma cells (Masuda et al., 2002), whereas its production in mice cells during viral pneumonia was found to proceed via inducible NO synthase (iNOS)-dependent NO overproduction (Akaike et al., 2003). *In vitro*, 8-nitropurine can be converted by a nucleophilic action of peroxynitrite to 8-oxopurine and this compound can be further oxidized by peroxynitrite to uric acid and its oxidation products (Lee et al., 2002).

As an important breakthrough, the formation of 8-nitro-cGMP, a nitrated cyclic nucleotide, was discovered in mouse macrophages with an active expression of iNOS. Importantly, 8-nitro-cGMP was found to possess the strongest redox-active and electrophilic properties among studied nitrated guanine derivatives (Sawa et al., 2007). Furthermore, 8-nitro-cGMP shows unique dual signaling functions, derived from its structural similarity to cGMP (i.e., activation of cGMP-dependent protein kinases) and its electrophilic properties due to the reactive nitro group (i.e., reactivity toward reduced thiols). *In vivo*, the formation of 8-nitro-cGMP does not proceed by the nitration of cGMP. 8-Nitro-cGMP is synthesized via the nitration of abundant GTP and subsequent action of guanylate cyclase on 8-nitroGTP to produce 8-nitro-cGMP (Fujii et al., 2010; Kunieda et al., 2015). Moreover, higher levels of 8-nitro-cGMP (≥40 μM) compared to cGMP levels (4.6 μM) were detected, uncovering 8-nitro-cGMP as the major intracellular cyclic nucleotide. After myocardial infarction, the 8-nitro-cGMP formation did not occur in mouse hearts deficient in iNOS, confirming the essential role of iNOS-derived NO for 8-nitro-cGMP formation (Nishida et al., 2012). Superoxide produced in mitochondria was identified as a determinant of 8-nitro-cGMP synthesis, whereas peroxynitrite as the molecular species involved in the reaction mechanism of guanine nitration; however, nitrite with H_2_O_2_ and myeloperoxidase can also nitrate guanine nucleotides in particular cellular environments (Ahmed et al., 2012). In LPS-treated rat glioma cells, the direct conversion of cGMP to 8-nitro-cGMP appeared unlikely, as the intracellular cGMP concentrations were one order of magnitude lower than 8-nitro-cGMP and inhibitors of soluble guanylate cyclase suppressed intracellular 8-nitro-cGMP generation.

## Metabolism of Nitrated Nucleotides

In contrast to cGMP, 8-nitro-cGMP is not degraded by the hydrolytic activity of phosphodiesterases (Sawa et al., 2013). The relative stability in the cellular environment presents a well-documented chemical feature of 8-nitro-cGMP, which enables to maintain its signaling functions. 8-nitro-cGMP shows electrophilicity much lower than other cellular electrophiles like unsaturated aldehydes and fatty acids, or nitroalkenes originating from lipid nitration. Consequently, the reaction rate of 8-nitro-cGMP and GSH thiol group is 20–10,000 times slower compared to these electrophiles (Sawa et al., 2010). Due to its relative chemical stability, 8-nitro-cGMP is expected to occur at noticeable concentrations even under high levels of reduced GSH. Moreover, several tested isoforms of glutathione transferases did not show any catalytical action to accelerate 8-nitro-cGMP degradation via its conjugation with GSH (Akaike et al., 2013). Nevertheless, it should be noted that 8-nitro-cGMP was unstable in degassed neutral phosphate buffer upon irradiation with the blue light (400 ± 16 nm) and decomposed to 8-nitrosoguanine and an open form of ribonolactone, with a half-life of 67.4 ± 1.8 min (Samanta et al., 2014). However, the biological relevance of light-driven 8-nitro-cGMP decomposition has not been so far addressed *in vivo*.

Metabolic fate of 8-nitro-cGMP in cell culture was assayed using stable ^18^O-labeled compound and LC-MS analysis. A novel nucleotide derivative, 8-amino-cGMP, was identified together with the S-guanylation products of 8-nitro-cGMP reaction with glutathione or cysteine (Saito et al., 2012). Immunochemical study based on prepared 8-amino-cGMP antibodies corroborated that the catabolism of 8-nitro-cGMP in LPS-triggered mouse macrophages proceeds to the formation of 8-amino-cGMP. Surprisingly, isotope-labeled 8-amino-cGMP was further converted to unmodified cGMP, suggesting oxidative modifications like guanine nitration and reducing pathways such as cGMP production would operate simultaneously during oxidative stress.

Hydrogen sulfide (H_2_S) belongs to reactive sulfur species with recognized signaling role across a wide range of organisms. In animals, H_2_S biosynthesis is controlled by two crucial enzymes: cystathionine β-synthase (CBS) and cystathionine γ-lyase (CSE). Knockdown of CBS and CSE resulted in elevated 8-nitro-cGMP concentrations, indicating that its activity might be regulated by sulfur species (Nishida et al., 2012). 8-mercapto-cGMP (8-SH-cGMP) was discovered by LC-MS analysis as a plausible product of S-guanylation reaction of 8-nitro-cGMP with H_2_S in mammalian cells. Treatment of 8-SH-cGMP *in vitro* with H_2_O_2_ or RNS provided intact cGMP. Thus inside cells, both 8-SH-cGMP and 8-amino-cGMP can be transformed into cGMP. However, the role of H_2_S in the formation of 8-SH-cGMP was later put to the question because of the *in vitro* reaction of 8-nitro-cGMP with the sulfide anion generates mainly 8-amino-cGMP (Terzič et al., 2014). Thus, endogenous H_2_S might act as a reductant in the transformation of 8-nitro-cGMP to 8-amino-cGMP; however, key roles of reactive hydropersulfides and related polysulfides in redox signaling and modifications of protein cysteines have been currently recognized (Akaike et al., 2017; Fukuto et al., 2018). In mice, hydropersulfides mitigated chronic heart failure after myocardial infarction, and this cardioprotective effect was mediated by repression of H-Ras pathway triggered by electrophilic action of 8-nitro-cGMP as a redox messenger for NO and ROS signaling. Hydropersulfide was shown to effectively thiolate cellular electrophiles, represented by 8-nitro-cGMP, indicating that electrophile thiolation can be considered a singular mechanism within ROS signaling and regulation of intracellular redox environment (Akaike et al., 2013). Later investigations revealed that CBS and CSE produce persulfide species showing higher nucleophilicity compared to H_2_S. Persulfides of cysteine and glutathione are namely produced and react with 8-nitro-cGMP to substitution products, which are then converted to 8-SH-cGMP by a thiol-disulfide exchange (Ida et al., 2014). The biological relevance of 8-SH-cGMP is indicated by the fact that it was recognized as the most abundant cGMP derivative in several mouse organs (Ida et al., 2014). Certainly, elucidation of redox signaling mechanisms of reactive persulfides counting low-molecular thiols and proteins together with protein S-guanylation opens a new era of redox biology, physiology, and pathophysiology (Kasamatsu et al., 2016), which awaits its investigation and recognition in plant sciences.

## Biological Activities of Nitrated Nucleotides

In early studies, nitrated derivatives of guanine or guanosine were considered rather as markers of nitrosative damage occurring in cells under stress conditions. Important redox-active features of 8-nitroguanosine, including generation of superoxide catalyzed by NADPH-cytochrome P450 reductase and NOS isoenzymes, were reported (Sawa et al., 2003). Soon after, 8-nitroguanosine was demonstrated to induce mutagenesis in animal cell culture (Yoshitake et al., 2004). Increased production of ROS and RNS was implicated in the development of lung cancer mediated by nitrosative and oxidative DNA modifications. Nitrosative stress associated with 8-nitroguanine generation results in lung epithelial injury in idiopathic pulmonary fibrosis (Terasaki et al., 2006). Oxidized and nitrated guanine derivatives were detected in cell cultures, tissues and organs from humans with degenerative diseases, cancer, viral pneumonia and other inflammatory conditions (Ohshima et al., 2006).

Later experiments evidenced biological activities and signaling functions of 8-nitro-cGMP were in major extent mediated by a PTM of protein thiols termed S-guanylation (Ihara et al., 2011; Nishida et al., 2016). Mechanisms of regulation of S-guanylation as protein PTM are actually not fully understood. It needs clarification if intracellular levels and distribution of NO and ROS may explain the observed site- and time-specific modulations of S-guanylation. S-guanylation, proceeding by a nucleophilic attack of the nitro group on protein cysteines, is considered an irreversible thiol modification. It is noteworthy that a similar replacement of the nitro group with thiol had not been reported previously. The reactivity of each cysteine residue varies considerably depending on its surrounding chemical and steric environment. The values of cysteines pKa in the target protein are affected by neighboring amino acid residues. Cysteine residues with lower pKa dissociate to sulfur anions that show higher reactivity with 8-nitro-cGMP. Basal levels of protein S-guanylation occurring in physiological conditions are elevated by inflammatory conditions. Due to the presence of numerous reactive cysteine residues, guanylation of protein Keap1 (Kelch-like ECH-associated1) was observed to occur even under a high excess of reduced glutathione (Sawa et al., 2007).

The discovery of new S-guanylated proteins provided further hints to biological roles of 8-nitro-cGMP. Protocols for S-guanylation proteomics have been developed and used to analyse the regulatory roles of protein S-guanylation in mitochondrial ROS export in animal cells stimulated with LPS or cytokines (Rahaman et al., 2014). S-guanylation of two key cysteine residues, Cys160 and Cys257, in heat-shock protein 60 controls the opening of mitochondrial permeability transition pore and export of mitochondrial ROS into the cytosol. In mice, increased levels of 8-nitro-cGMP following myocardial infarction suggested its role in the pathogenesis of heart failure (Nishida et al., 2012). In this experimental model, 8-nitro-cGMP acts as a physiological ligand activating H-Ras protein, when S-guanylation at Cys184 drives H-Ras translocation to non-raft membrane domains and activation of its downstream signaling pathways.

Hepatocyte growth factor ameliorated high glucose-induced oxidative stress in rat mesangial cells by elevated NO-dependent 8-nitro-cGMP production (Guoguo et al., 2012). 1-nitro-2-phenylethane restricted taurocholate-induced cell death in pancreatic cells by increasing 8-nitro-cGMP production mediated by the sGC (Cosker et al., 2014). 8-nitro-cGMP also showed significant cytoprotective capacity in dopaminergic neurons by S-guanylation leading to induction of hem oxygenase 1 (HO-1) (Kurauchi et al., 2013; Kasamatsu et al., 2014). Similarly, HO-1 activated by NO-dependent 8-nitro-cGMP production participates in macrophage defense to *Salmonella* infection (Zaki et al., 2009). In studies of the Alzheimer disease, 8-nitro-cGMP guanylated cysteine residues in two tau proteins, which eliminated their capacity to form tau aggregates (Yoshitake et al., 2016). Cell exposure to the exogenous electrophile methylmercury, which triggers NO and ROS signaling, elevated intracellular 8-nitro-cGMP, depleted reactive persulfides and 8-SH-cGMP, increased S-guanylation and activation of H-Ras leading to damaged cerebellar neurons (Ihara et al., 2017).

8-nitro-cGMP was found to S-guanylate thiol groups of cGMP-dependent protein kinases (PKG), the primary sensor proteins of intracellular cGMP known to control an array of cellular reactions (Ahmed et al., 2017). S-guanylation of PKG occurs specifically at two susceptible residues Cys42 and Cys195 among 11 cysteine residues of PKG. Notably, S-guanylation of Cys195, positioned in the cGMP binding domain, results in an enduring activation of the enzyme activity. In mice, S-guanylation of PKG was observed without any treatment and was strongly increased by LPS treatment (Akashi et al., 2016). It was suggested that degradation of S-guanylated proteins might proceed by autophagy (Taguchi et al., 2012). Interestingly, induction of autophagy was revealed as another important function of 8-nitro-cGMP in animals (Ito et al., 2013; Rawet-Slobodkin and Elazar, 2013; Abada and Elazar, 2014). Autophagy can efficiently function as an innate defense to pathogen infection (Nakagawa et al., 2004; Mizushima and Komatsu, 2011). 8-nitro-cGMP supported autophagic elimination of infecting group A Streptococci in mice macrophages, where autophagosome-encapsulated pathogens showed higher S-guanylation compared to pathogens in the cytosol, indicating S-guanylation might be exploited to tag bacteria or disease-related fragments and cellular debris for autophagic removal (Ito et al., 2013; Takahashi et al., 2019). Inversely, bacteria are capable to interfere with autophagy-mediated pathogen clearance via production and release of reactive persulfides which inhibit autophagy signaling by the degradation of 8-nitro-cGMP in host cells (Khan et al., 2018).

## Biological Functions in Plants

The currently accumulated evidence has unraveled roles of cyclic nucleotides including cGMP in multiple plant physiological processes, including plant growth and development from germination to flowering (Lemtiri-Chlieh et al., 2011; Gehring and Turek, 2017). Compared to animals, the reaction pathways and signaling function of cGMP are not completely described and no homologs of animal cGMP-producing enzymes have been identified in higher plants; however, NO-dependent cGMP pathway participates in multiple signaling mechanisms in plants, namely in the regulation of stomata opening and defense response to pathogenic challenge (Gross and Durner, 2016). However, the molecular mechanisms of cGMP signal transduction to cellular effectors, including cGMP-dependent kinases G, are still poorly characterized (Świeżawska et al., 2018). Recent reports described the identification of cGMP-dependent protein kinase with a role in mediating gibberellin responses in rice (Shen et al., 2019) and a plant cGMP-activated phosphodiesterase involved in the UVA-induced cGMP degradation in Arabidopsis stomata (Isner et al., 2019). Signaling pathways involved in the regulation of stomatal movements by external stimuli and phytohormones comprise, beside cGMP, diverse components like NO, ROS, cytosolic pH, calcium ions and phospholipids (Daszkowska-Golec and Szarejko, 2013; Gayatri et al., 2013; Agurla et al., 2014; Agurla and Raghavendra, 2016). Moreover, protein PTMs by phosphorylation and redox modifications have key functions in the regulation of stomatal movement. These modifications control molecular components of signal perception, second messenger production and downstream events within guard cell signaling (Balmant et al., 2016). NO fulfils crucial functions in modulation of stomatal movement, namely in abscisic acid-induced stomatal closure. NO is involved in stomatal closure induced by H_2_S, polyamines and methyl jasmonate (Sun et al., 2019) as well as microbe-associate molecular patterns (Ye and Murata, 2016). Interactions and cross-talk of the signaling pathway of NO and H_2_S are quite complex and both gasotransmitters regulate stomatal movement independently or in united action, in ABA-dependent signaling cascades or ABA-independent mechanisms (Scuffi et al., 2016).

**FIGURE 1:**
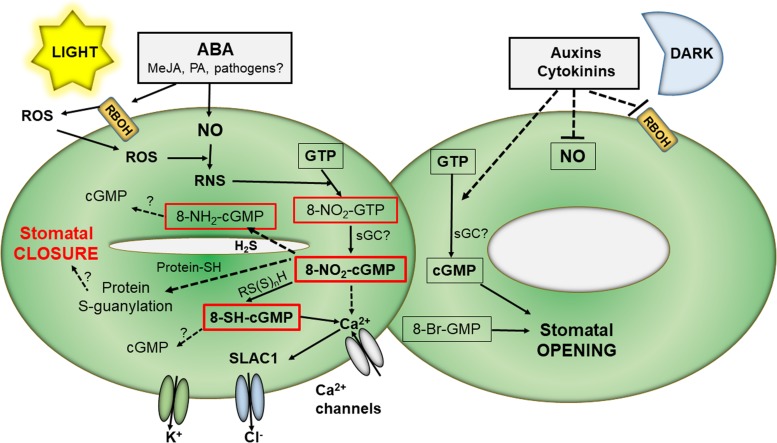
Overview of 8-Nitro-cGMP reaction pathways in stomata signaling. In the light, external stimuli like ABA induce in stomata guard cells increased NO synthesis as well as activation of ROS production by membrane NADPH oxidase. ROS react with NO to form RNS which can nitrate GTP to 8-nitro-GTP which is then converted to 8-nitro-cGMP, possibly by action of sGC. 8-Nitro-cGMP, and/or 8-SH-cGMP formed by its reaction with reactive persulfides, trigger elevated cytoplasmic Ca^2 +^ levels and subsequently activation of slow anion channels leading to stomatal closure. In the dark, other plant hormones, including cytokinins and auxin, decreases both NO and ROS levels resulting in increased cGMP and stomatal opening. ABA, abscisic acid; cGMP, 3′,5′-cyclic guanosine monophosphate; GTP, guanosine triphosphate; MeJa, methyl jasmonate; PA, polyamines; RBOH, NADPH oxidase; RNS, reactive nitrogen species; ROS, reactive oxygen species; RS(S)_n_H, persulfides; sGC, soluble guanylate cyclase; SLAC, slow anion channels.

Intriguing observations that cGMP was needed but not sufficient for stomatal closure induced by ABA were in agreement with experiments using Arabidopsis mutants in cGMP-dependent calcium channels which were not impaired in ABA-triggered stomatal closure (Dubovskaya et al., 2011; Wang et al., 2013). Later, the occurrence and functional implications of 8-nitro-cGMP in Arabidopsis stomatal guard cells were discovered (Joudoi et al., 2013). cGMP and 8-nitro-cGMP appear to show contrasting impact on stomata function: 8-nitro-cGMP induced stomatal closure in the light, whereas 8-Br-cGMP, an extensively utilized cGMP analog, did not. On the contrary, 8-Br-cGMP but not cGMP induced stomata opening in the dark. 8-nitro-cGMP signaling is mediated by modulation of Ca^2+^, cyclic adenosine-5′-diphosphate ribose, and the SLAC1 (*SLOW ANION CHANNEL1*) channels. Increased levels of RNS, produced by ROS and NO reactions, resulted in 8-nitro-cGMP production triggering increased levels of cytosolic Ca^2+^ which activated the SLAC1 anion channels to promote stomata closure. In the following study, the 8-nitro-cGMP metabolite 8-SH-cGMP was also observed to induce closure of stomata pores (Honda et al., 2015). Nevertheless, the participation of protein S-guanylation in 8-SH-cGMP signaling was not evidenced (Figure 1).

It can be expected that similar to animal models (Sawa et al., 2007), 8-nitro-cGMP retains the capacity to activate plant cGMP-dependent protein kinases. In animal cells, intracellular 8-nitro-cGMP levels are supposed to be comparable or higher than cGMP levels during infection or inflammatory conditions (Fujii et al., 2010; Kunieda et al., 2015). Therefore, the role of 8-nitro-cGMP and its metabolites 8-amino-cGMP and 8-SH-cGMP as PKs activators in plant physiology and stress responses requires further investigation.

In plants, autophagy mediates selective destruction of viruses as well limits infection by bacterial and filamentous pathogens. Emerging evidence indicates that autophagy is a key regulator of plant innate immunity and contributes with both pro-death and pro-survival functions to antimicrobial defenses, depending on the pathogenic lifestyle (Hofius et al., 2017; Leary et al., 2019). The role of H_2_S in regulating plant autophagy has been recently demonstrated (Gotor et al., 2013; Laureano-Marín et al., 2016). Sulfide, but no other molecules such as sulfur-containing molecules or ammonium, was able to inhibit autophagy machinery induced in *A. thaliana* roots under nitrogen deprivation via a redox-independent mechanism. H_2_S-mediated signaling in autophagy has been suggested to be mediated by post-translational modifications of the enzymes involved in the ubiquitinylation process or of other proteins involved in the initiation and completion of the autophagosome. The action of H_2_S may include protein S-persulfidation at the reactive cysteine residue(s) of the target proteins. Polyamine spermine was reported to induce autophagy in plants, mediated by increased generation of ROS and NO required for effective triggering of autophagy (Dmitrieva et al., 2018). Combined effects of NO and ROS is required for autophagy and necrotic HR cell death induced by *Alternaria alternata* toxin in tobacco BY-2 cells (Sadhu et al., 2019). In Arabidopsis, glycolate oxidase activity is induced by avirulent Pst DC3000 AvrRpm1 and this response is suppressed by a synergic action of NO and cGMP. The enzyme activity *in vitro* was inhibited by combined treatment with cGMP and NO donor, but it is not known if this effect was mediated by 8-nitro-cGMP (Donaldson et al., 2016). Given the widely demonstrated role of 8-nitro-cGMP in autophagy processes in animals, this deserves further investigations on plant cells and components both *in vitro* and *in vivo*.

## Future Perspectives

To date, only two scientific reports investigated the functions of 8-nitro-cGMP and 8-SH-GMP in plant cell signaling and regulation. Further progress in this field may be hampered by several experimental obstacles: the pure chemical standards of described compounds are not commercially available, their synthesis requires dubious purification and analytical techniques requires mass-spectrometry instrumentation. A reporter system has been developed for specific and sensitive detection of cyclic nucleotides including cGMP in bacteria and plants; however, if this system shows response also to cGMP derivatives has not been tested (Wheeler et al., 2013). It should be also considered that available commercial antibodies might lack required specificity, i.e., to show affinity to 8-nitroguanine and 8-nitroguanosine but also 8-nitroxanthine (Sawa et al., 2006). Furthermore, very limited information exists on how and in what extent S-guanylation might function as protein PTM in plant cells. As discussed above, it requires further investigations to clarify if cysteine pKa may control the required specificity of S-guanylation targets in cellular signaling and autophagy; thus, the nature of putative enzymes that might catalyze S-guanylation continues an essential point for the advances in this research area both in plants and animals. Moreover, a combined application of available transcriptomic, proteomic and metabolomics tools to plant cells treated with 8-nitro-cGMO and its metabolites can provide new insights in their role in NO- and ROS-dependent plant signaling.

## Author Contributions

MP and LL designed the manuscript. MP wrote the initial version of the manuscript. LL read and corrected the final version.

## Conflict of Interest

The authors declare that the research was conducted in the absence of any commercial or financial relationships that could be construed as a potential conflict of interest.
